# Corticocerebellar Effective Connectivity During Adapting to vs. Ignoring Delayed Visual Movement Feedback

**DOI:** 10.1111/ejn.70397

**Published:** 2026-03-17

**Authors:** Zhenyu Wang, Jakub Limanowski

**Affiliations:** ^1^ Institut für Psychologie Universität Greifswald Greifswald Germany

**Keywords:** action, cerebellum, delay, forward model, visuomotor adaptation

## Abstract

Internal models in the brain may enable flexible action control by calculating estimates of the body's state and predictions of the sensory consequences that its actions will produce. These processes are thought to be implemented by interactions among cortical and subcortical brain regions including the cerebellum. During a virtual reality based hand‐target matching task, in which delayed visual movement feedback was behaviorally relevant (i.e., requiring visuomotor adaptation) or irrelevant (i.e., needed to be ignored), we had observed increased hemodynamic responses in the cerebellum (left Crus I/right Lobule VI), V5, and intraparietal sulcus during the adaptation condition. These activity changes suggested processes specific to delay‐dependent adaptation. Here, we used dynamic causal modeling (DCM) to test if these regional activity changes could be explained in terms of task (i.e., adaptation)‐dependent between‐region connectivity changes. During visuomotor adaptation, DCM revealed an increased excitatory influence of the right cerebellum (Lobule VI) on bilateral V5 (and on the IPS), and an increased mutual excitation among the right cerebellum and the left IPS. Our results support the idea that the communication of cerebellar predictions to the cortical visuomotor network underlies visuomotor adaptation.

## Graphical abstract

We applied dynamic causal modeling to fMRI data acquired during a virtual reality based visuomotor task. Visuomotor adaptation, compared with no‐adaptation, was associated with an increased excitatory influence of the right cerebellum on bilateral V5 and the IPS. These results suggest that, during visuomotor adaptation, the cerebellum issues predictions of sensory action consequences to visuomotor cortical hierarchies.

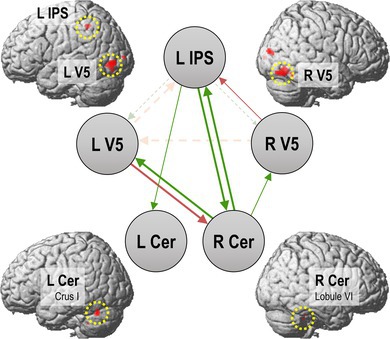

**Abbreviations**
BMA: Bayesian model averaging
BMR: Bayesian model reduction
BOLD: blood‐oxygen‐level‐dependent
Cer: cerebellum
DCM: dynamic causal modeling
fMRI: functional magnetic resonance imaging
GLM: general linear model
IPS: intraparietal sulcus
MNI: Montreal Neurological Institute
PEB: parametric empirical Bayes
PPC: posterior parietal cortex
RH: real hand
ROI: region of Interest
SPM: statistical parametric mapping
VH: virtual hand

## 1 Introduction

Online motor control relies on flexible internal models in the brain, which, among other things, must entail accurate estimates of the state of the body, and accurate predictions of the sensory consequences that its actions will produce (Wolpert and Flanagan 2001; Shadmehr and Krakauer 2008). Sensory action consequences can be predicted by so‐named “forward models” based on copies of motor signals (Miall et al. 1993; Wolpert, Goodbody, et al. 1998; Wolpert, Miall, et al. 1998; Desmurget and Grafton 2000). Updating these forward models by sensory prediction errors is essential for adapting to novel sensory movement feedback (Galea et al. 2011; Izawa et al. 2012; Danckert et al. 2008; Luauté et al. 2009; Tseng et al. 2007). Neurophysiological, brain imaging, and clinical studies suggest that such state estimation and forward modeling may be implemented by the cerebellum and the posterior parietal cortex (PPC, Blakemore et al. 1998; Desmurget et al. 1999; Miall et al. 1993; Wolpert, Goodbody, et al. 1998; Wolpert, Miall, et al. 1998; Leube 2003; Synofzik et al. 2008; Izawa et al. 2012; Manto et al. 2012). Besides changes in regional brain activity, several studies have observed changes in corticocerebellar connectivity during the prediction of sensory action consequences (Kilteni and Ehrsson 2020; Kellermann et al. 2012; Arikan et al. 2019; Tzvi et al. 2020).

We (Vigh and Limanowski 2025) recently reported increased PPC, occipitotemporal, and cerebellar blood‐oxygen‐level‐dependent signal (BOLD) in a task where participants had to adapt their hand movements to repeatedly varying visuomotor delays (i.e., temporal lags added to the visual movement feedback), compared with when they moved under identical visuomotor delays while ignoring them. Cerebellar (left Crus I/right Lobule VI) BOLD signal correlated with visuomotor delay more strongly in the adaptation task; i.e., when visual movement feedback (and its temporal congruence with muscle movements) was behaviorally relevant. These results suggested a network of brain areas specifically involved in predicting and processing visual movement feedback depending on its behavioral relevance.

Here, we subjected these data to dynamic causal modeling (DCM, Friston et al. 2003) to test whether the observed task‐dependent regional BOLD signal changes could be explained in terms of changes in interregional communication among these areas, potentially in line with the assumption of cerebellar (or parietal) sensory forward predictions.

## 2 Methods

### 2.1 Participants

We reanalyzed the fMRI data acquired by Vigh and Limanowski (2025). Twenty healthy, right‐handed participants (15 female, mean age = 28.1 years, range = 22–38, normal or corrected‐to‐normal vision) had taken part in the experiment, which had been approved by the ethics committee of the Technische Universität Dresden.

### 2.2 Experimental Task and Design

**FIGURE 1:**
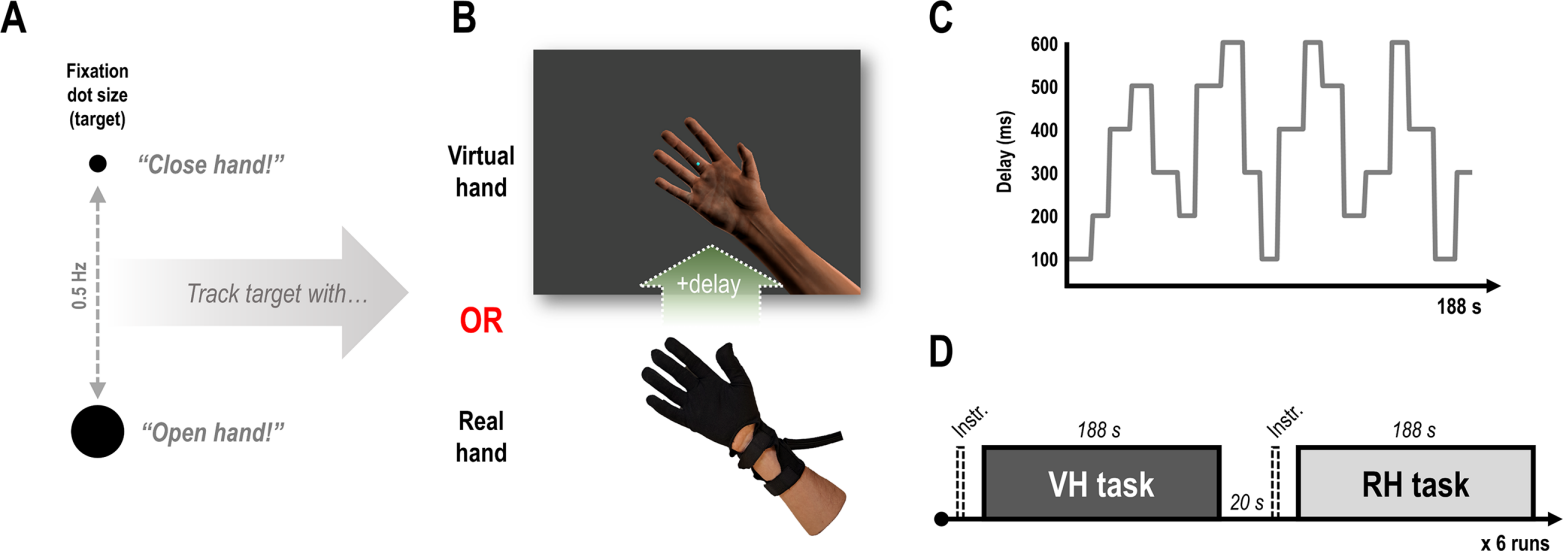
Experimental task and design. (A) The participants had to match the phase of an oscillating dot with recurrent right‐hand (open‐and‐close) movements. (B) Via an MR compatible data glove worn on their right hand, participants controlled a photorealistic virtual hand model. The virtual hand movements were permanently delayed with respect to the real hand movements. Thus, only one of the hands (virtual or real) could be aligned with the target oscillation at a time, while the other one consequently moved out of phase. (C) During the continuous movement task, the amount of delay added to the virtual hand movements varied between 100 and 600 ms in a roving oddball fashion. (D) Participants were instructed to match the target oscillation with the movements of either the virtual hand (VH) or their real, unseen hand (RH). Thus, participants either had to attend to, and try to adapt to the visuomotor delays (VH task), or to ignore them (RH task). Reprinted from Vigh and Limanowski (2025).

Participants had to perform a hand‐target phase matching task; i.e., they had to match the phase (0.5 Hz) of an oscillating fixation dot with right‐hand grasping movements (Figure 1A). Participants wore a data glove on their unseen hand, which transmitted finger flexion data to a virtual hand model presented on screen. Thus, the participants could move the fingers of the virtual hand (Figure 1B). During the continuous movement task (in blocks of 188 s, see below), the movements of the virtual hand were permanently delayed with respect to the actually executed hand movements; this implied a constant visuomotor and visuoproprioceptive mismatch. Crucially, participants were instructed to match the target oscillation with their unseen, “real” hand (RH) or with the virtual hand (VH) that was presented on screen. Note that, due to the delay added to the visual movements, only one of the two hands (VH or RH) could be aligned with the oscillating target at a time—the other hand would consequently move out of phase with the target. For instance, to align the virtual hand with the target, participants had to try to adapt their real hand movements (phase shift them to compensate for the delay); to align the real hand, participants had to ignore the visual feedback delay. Thus, the task instruction determined whether or not visual feedback delays were task relevant, and whether or not participants needed to try to adapt to them or ignore them. The amount of visual feedback delay was repeatedly varied between 100 and 600 ms in a roving oddball fashion, in pseudorandomized sequences, requiring repeated visuomotor adaptation (in the VH task, Figure 1C). Each of six delay sequences consisted of 94 movement cycles (188 s) and was used for the VH and RH task alike (Figure 1D).

As expected, our previous results (Vigh and Limanowski 2025) showed that participants significantly more strongly phase‐shifted their real hand movements depending on the amount of delay in the VH > RH task. Furthermore, the group‐level statistical parametric mapping (SPM) analysis of the fMRI data had identified several key regions that showed stronger activation and, partly, stronger delay‐related responses in the VH > RH task: Firstly, the left intraparietal sulcus (IPS) and the bilateral middle and inferior occipital gyri showing a significantly (*p*_*FWE*_ < 0.05) stronger BOLD signal during the VH > RH task. This suggested an involvement of these regions in visuomotor integration and related attentional control during the VH > RH task. Here we assign these occipital activations the label “V5,” as they very likely correspond to these motion sensitive areas. Secondly, we identified regions in the left (Lobule VIIa/Crus I) and right cerebellum (Lobule VI) showing an increased BOLD signal correlation with the parametric delay regressor in the VH > RH task; these regions also showed increased BOLD signal during the VH > RH task in a conjunction search (i.e., masking two contrasts with each other at *p* < 0.001, uncorrected). Thus, the cerebellar response increase was elevated during the VH > RH task in a delay‐dependent manner, suggesting a process related specifically to delay compensation and visuomotor adaptation. No other regions showed significant effects of the VH > RH task or delay regressor.

### 2.3 DCM Analysis

**FIGURE 2:**
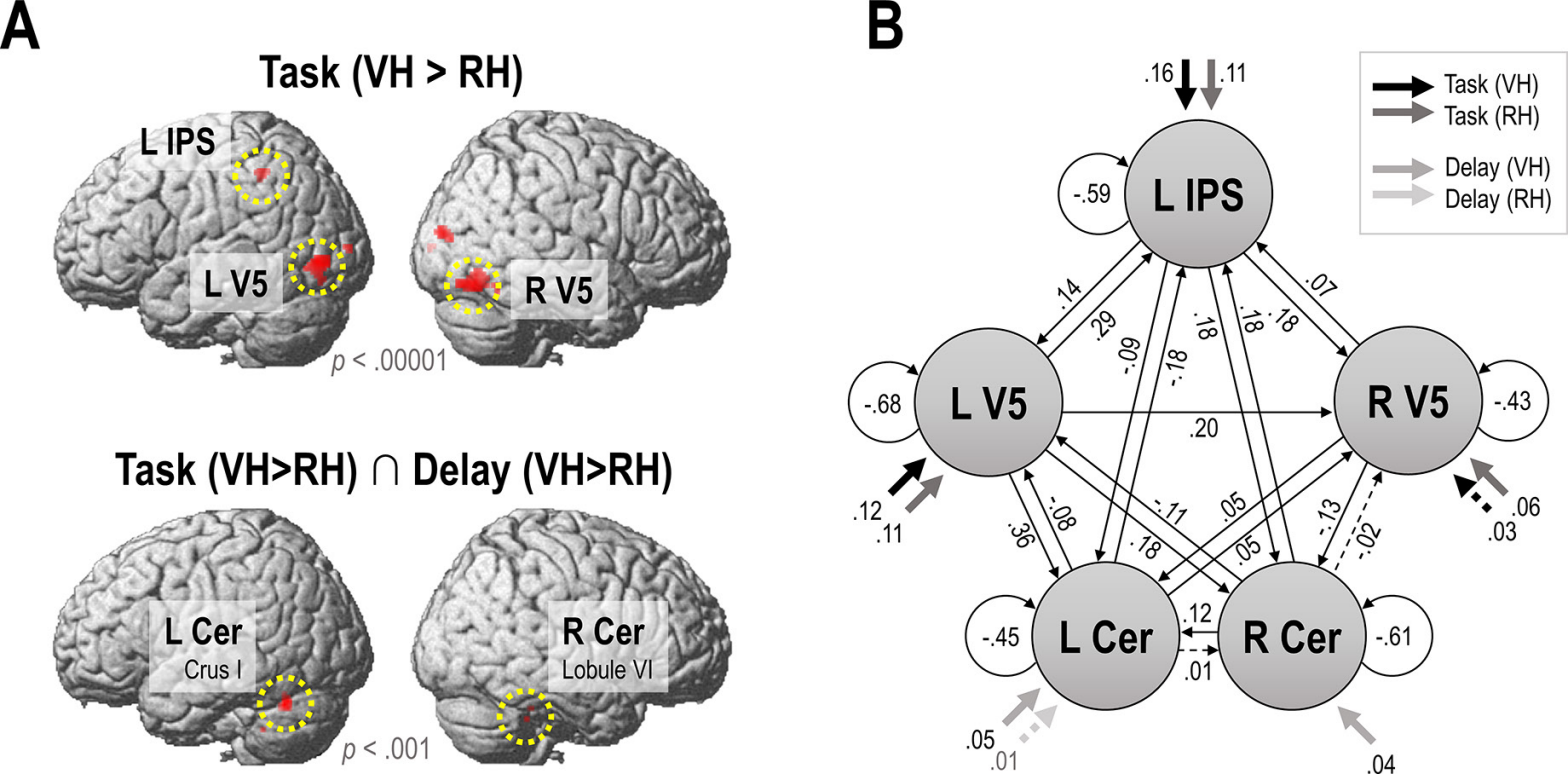
DCM architecture. (A) Based on the significant effects obtained from the group‐level SPM analysis, five nodes of interest (circled in yellow) were selected for the DCM analysis: The left IPS and bilateral V5 showed significantly increased activation during the VH > RH task (*p*_*FWE*_ < 0.05, render thresholded at *p* < 0.00001 for display purposes); the bilateral cerebellum (left Lobule VIIa/Crus I and right Lobule VI) showed a stronger activation by delays in the VH > RH task and an increased activation by the VH > RH task per se (*p* < 0.001, uncorrected). (B) DCM model architecture showing driving inputs (C matrix) and baseline network connectivity (A matrix) among the five nodes of the full model. Parameter estimates denote rates of change (in Hz). Only nonzero parameter estimates are shown. Dashed lines indicate connections with only weak evidence (posterior probability < 0.75).

We performed DCM in SPM12.5 (https://www.fil.ion.ucl.ac.uk/spm). The focus of our connectivity analysis was on explaining the stronger activations and delay correlations observed during the VH > RH task in terms of network interactions; i.e., changes in connectivity among our regions of interest (ROIs). The above five ROIs, the left IPS, bilateral V5, and the bilateral cerebellum, were selected as nodes for our DCM analysis (Figure 2A). For each ROI, we extracted the (slice‐acquisition delay corrected) BOLD signal time series, concatenating the six experimental runs, from 4‐mm radius spheres centered on the individual peak voxel (i.e., the strongest participant specific effect) within 10 mm of the significant group‐level effect (see above); i.e., using the contrast Task VH > RH for the IPS and V5s, and the contrast Delay VH > Delay RH for the cerebella. The mean MNI coordinates of the peak effects with associated standard deviations were left IPS (*x* = −38.5 ± 4.3, *y* = −42.1 ± 4.0, *z* = 51.9 ± 5.4); left V5 (*x* = −44.0 ± 4.4, *y* = −76.4 ± 4.1, *z* = −3.3 ± 4.3); right V5 (*x* = 42.0 ± 5.0, *y* = −70.1 ± 4.5, *z* = −16.4 ± 4.3); left cerebellum (*x* = −37.3 ± 4.9, *y* = −60.8 ± 4.7, *z* = −25.2 ± 4.7); right cerebellum (*x* = 37.1 ± 4.6, *y* = −40.7 ± 4.4, *z* = −31.4 ± 5.2). To reduce noise, we extracted the time series only from voxels showing the desired effect at a statistical threshold of *p* < 0.05, uncorrected (cf. Vossel et al. 2012); in some cases, we had to increase the statistical threshold to reveal significant voxels (for one participant's ROI, we extracted from the group peak as no significant effect was found within the search radius; cf. Zeidman, Jafarian, Corbin, et al. 2019). The time series were adjusted for effects of no interest (movement regressors and session means).

In DCM, effective connectivity implies changes in the activity in one region caused by activity in another region (i.e., parameter estimates are rates of change in Hz), but it does not necessarily imply that these changes are mediated by direct (monosynaptic) structural connections (Friston et al. 2003, 2011; Friston 2011). Therefore, for the intrinsic connectivity of our model (A matrix), we allowed bilateral connections between all nodes of the network; i.e., each node could, in principle, affect activity in all other nodes (potentially, via unmodelled other regions). Thus, we also allowed for ipsilateral corticocerebellar and for cerebellar interhemispheric coupling (which has been reported previously, e.g., Kipping et al. 2013; Karavasilis et al. 2019; Pollok et al., 2006; Haihambo et al. 2025). Each node also received an inhibitory “self”‐ connection.

For assigning driving inputs (C matrix), we included a separate model comparison. Note that driving inputs could be thalamic, but may also stem from other, unmodelled brain regions such as primary visual or (pre)motor cortices. I.e., we assumed that V5 received visual inputs of the task, while the IPS and the cerebella could, potentially, receive nonvisual inputs, e.g., from somatosensory or motor regions (cf. Bencivenga et al. 2021; Tzvi et al. 2020). Thus, in the full model, we allowed the driving input of the VH and RH task, respectively, to each node; and compared this model with two alternatives, one receiving task inputs at V5 and IPS, and one receiving those at V5 and the cerebella. Driving inputs of the delay effects (i.e., regressors consisting of the parametric delay effect in the VH and RH task, respectively) were allowed to the bilateral cerebellum, because only these nodes showed a main effect of delay in the GLM analysis. The model with task inputs to the IPS and bilateral V5, and delay inputs to the cerebella, outperformed the other models with 100% posterior probability. This architecture was therefore chosen for estimating the full model, upon which the modulatory effects were calculated.

Figure 2B shows the nonzero parameter estimates of the intrinsic connectivity of the network at baseline, and the effects of the driving inputs (in the RH task, the driving delay effect on the right cerebellum was estimated to be zero, i.e., absent). We then defined two modulatory inputs (B_1_ and B_2_ matrices): of the VH and the RH task, respectively. The inputs were not mean centered, so the B matrix (modulatory) parameters can be interpreted as increases or decreases in connection strength, during the VH or RH task, compared with baseline activity captured by the A matrix. We used DCM “network discovery” (Friston et al. 2011); i.e., we did not constrain our modulatory connections a priori but initially allowed the modulations to act upon all between‐region connections in the full model. The full model was first estimated (inverted) for each participant; on average, these models explained 23.8% of the experimental variance. To determine which B matrix connectivity parameters explained the observed BOLD signal time differences, we then used automatic Bayesian model reduction (BMR; Friston et al. 2016) within a parametric empirical Bayesian (PEB; Zeidman et al. 2019, 2019) approach, where a “full” model is estimated (inverted) for each participant and inference is subsequently performed on reduced models. In the BMR, reduced models consist of various different combinations of B matrix parameters switched off; the automatic search algorithm iteratively discards those parameters that do not contribute meaningfully to model evidence—by evaluating each parameter's posterior probability—until discarding further parameters decreases model evidence (Zeidman, Jafarian, Seghier, et al. 2019). In other words, a model with the retained parameters (modulated connections) explains the data (BOLD signals) better than a model without them. Then, we calculated the Bayesian model average (BMA) over the 256 models of the final iteration of the search; i.e., the retained parameter estimates reflect the averages from different models weighted by the models' respective posterior probabilities (Penny et al. 2010). We thresholded the results of this BMA at a posterior probability of > 0.75 to highlight parameters with positive evidence for a contribution to model evidence; i.e., a model with this parameter would notably outperform a model without it. For completeness, we report all retained parameters in Table 1, including those with posterior probabilities < 0.75. Finally, to illustrate the strength of the notable cerebellar connectivity differences between the VH and RH task, we computed Bayesian contrasts (cf. Dijkstra et al. 2017).

**TABLE 1:** Bayesian model averages of parameters retained in the BMR with associated posterior probabilities in brackets (unthresholded).

Anatomical connection	B1: Modulation by VH task (Hz)	B2: Modulation by RH task (Hz)
L IPS—L V5	0.13 (58)	0.21 (.72)
L IPS—R V5	0.22 (.81)	0.14 (.58)
L IPS—L Cer	0.12 (.58)	—
L IPS—R Cer	0.39 (1)	0.24 (1)
L V5—L IPS	−0.40 (1)	−0.39 (1)
L V5—R V5	—	—
L V5—L Cer	—	—
L V5—R Cer	−0.25 (1)	—
R V5—L IPS	−0.12 (0.58)	—
R V5—L V5	−0.28 (1)	−0.25 (1)
R V5—L Cer	—	—
R V5—R Cer	—	—
L Cer—L IPS	—	—
L Cer—L V5	—	—
L Cer—R V5	—	—
L Cer—R Cer	—	−0.11 (.56)
R Cer—L IPS	0.40 (1)	0.29 (1)
R Cer—L V5	0.38 (1)	—
R Cer—R V5	0.15 (0.63)	—
R Cer—L Cer	—	—

## 3 Results

**FIGURE 3:**
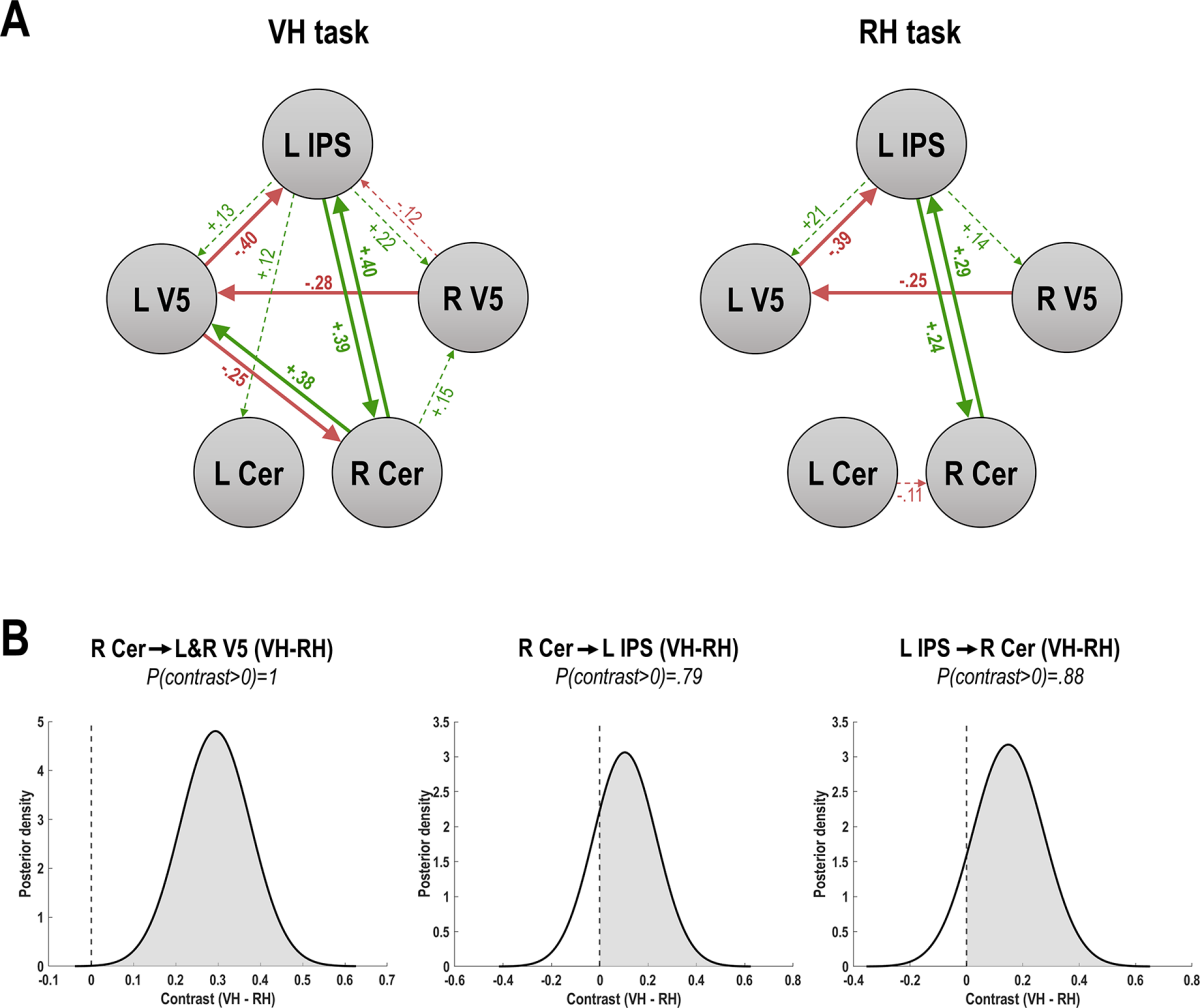
Task‐dependent connectivity changes. (A) Results of the BMR, showing the retained modulatory parameters; i.e., the task‐dependent changes in connectivity (as rates of change, in Hz) of the winning (reduced) model. Green and red colors reflect positive and negative modulations, respectively; thick lines indicate estimates with a posterior probability > 0.95, dashed lines those with weaker evidence. The parameter estimates are Bayesian model averages over the final model space, reflecting the increases or decreases in connectivity under each task. See Table 1 for details. (B) Posterior densities of Bayesian contrasts over parameter estimates (VH task—RH task) showed positive evidence for that the connections from the right cerebellum (Cer) to bilateral V5, and the mutual connections between the right cerebellum and the left IPS were more excitatory in the VH task compared with the RH task (indicated by positive values of the posterior means).

BMR (Figure 3A) retained several modulatory parameters, i.e., increases or decreases in connectivity strength that contributed to model evidence, in each task: Both tasks were associated with an increased excitatory influence of the right cerebellum on the left IPS, of the IPS on the right cerbellum and bilateral V5, an inhibitory influence of left V5 on the IPS, and of right on left V5. See Table 1.

Crucially, the cerebellar connections showed marked differences between tasks: There was an excitatory influence of the right cerebellum onto both V5s in the VH, but not the RH task (where these connections were removed, i.e., set to 0 by the BMR). Furthermore, the mutual excitatory influence of the right cerebellum on the left IPS, and vice versa, was likewise increased in the VH>RH task. Bayesian contrasts (Figure 3B) over the corticocerebellar connectivity parameters showed that these differences had high posterior probabilities of being nonzero.

## 4 Discussion

Our DCM results highlight the role of network interactions of the (right) cerebellum in a task involving adaptation to delayed visual movement feedback (VH task), compared with moving while ignoring those delays (RH task).

Notably, while delays activated the bilateral cerebella in the VH but not (or only weakly) in the RH task, only the connectivity of the right cerebellum showed different modulatory effects between tasks. The peak BOLD signal differences were located in the left Lobule VIIa/Crus I and the right Lobule VI, respectively, two functionally distinct regions (Ito 2008; O'Reilly et al.  2010; Diedrichsen et al. 2019; Saadon‐Grosman et al. 2024). Activation of Crus I has been linked to cognitive functions including working memory and the encoding of contextual rules (e.g., Buckner et al. 2011; Guell et al. 2018; Ma et al. 2023). In contrast, activation of Lobule VI has been observed predominantly in sensorimotor and spatial tasks, notably including visuomotor adaptation (Stoodley & Schmahmann 2009; Tzvi et al. 2020, 2022; cf. Bernard et al 2012; Kilteni & Ehrsson 2020).

Our DCM results support this functional distinction, and specifically imply the (right) cerebellar Lobule VI in the communication of predictive signals for visuomotor adaptation: Firstly, the increased excitatory effect of the right cerebellum/Lobule VI on bilateral V5 only in the VH task indicated a selectively increased sensitivity of V5 to cerebellar signals when adaptation to the visual feedback delays was required. We propose this suggests an enhanced communication of cerebellar predictions of visual movement consequences, which required repeated updating in the VH (adaptation) task, but were irrelevant in the RH task. This “top‐down” communication of predictions to visual areas suggest the cerebellum at a hierarchically higher level, in line with previous studies (Kellermann et al. 2012).

Secondly, the right cerebellum had an increased excitatory effect on the IPS in both tasks, and vice versa. Notably, both excitatory connections were stronger in the VH than in the RH task. In other words, the right cerebellum and the left IPS mutually increased their positive influence over each other in the adaptation task. Following the assumed close interaction of the PPC and the cerebellum during online action control, this result could suggest the recurrent exchange of (updated) state estimates. Thus, the cerebellum could receive parietal signals and send the necessary updates back. Potentially, the slight excitatory influence of the IPS over the left Crus I in the VH task could, in contrast, indicate the communication of different control parameters of the adaptation task (e.g., related to attentional context or higher level rules).

Furthermore, V5 had inhibitory effects on other nodes, which partly differed between tasks. An increased inhibition of the PPC or the cerebellum by higher level visual regions has been observed in other DCM studies, e.g., during attention to motion, visual perception, or action observation (Kellermann et al. 2012; Dijkstra et al. 2017; Li et al. 2025). Kellermann et al. 2012 proposed a reduced parietal sensitivity to bottom‐up prediction errors from V5 could augment visual motion prediction. A similar interpretation could be applied to our results, as the IPS was inhibited by right V5 in the VH but not the RH task. The right cerebellum was, similarly, inhibited by left V5 only in the VH task. Thus, the influence of prediction errors from delayed visual motion could have been attenuated in the VH task, in favor of predictive processing in parietal and cerebellar models.

It should be noted that there is some uncertainty associated with assigning anatomical labels in DCM, where time series are averaged over many voxels in ROIs around individual peak effects. Therefore, the labels “Crus I” and “Lobule VI” assigned to the cerebellar nodes should be interpreted with the usual caution. It should, furthermore, be noted that the potentially indirect relationship between structural and effective connectivity (see above) and the restriction of our model to nodes showing significant BOLD signal differences leaves open the possible involvement of other (e.g., visual or motor) areas or processes (e.g., attentional). Our results should hence be interpreted together with previous findings of cerebellar connectivity with premotor regions during visuomotor (de)adaptation (Tzvi et al. 2020), changes in connectivity of somatosensory vs. visual regions during intersensory conflicts (Limanowski and Blankenburg 2015; Limanowski and Friston 2020; Pamplona et al. 2025), and with the observed effects of prediction and attention on early visual cortical connectivity (Kok et al. 2012; Kellermann et al. 2017; cf. Büchel et al. 1998), in the dorsal or ventral streams (Vossel et al. 2012, 2015; Zbären et al. 2024), or the motor system (Bencivenga et al. 2021). Our results add to these previous findings by showing that the cerebellum (right Lobule VI) conveys predictive signals to visual (and parietal) regions during visuomotor adaptation.

## Author Contributions

**Z.W.:** formal analysis (equal), visualization (equal), writing – review and editing (supporting). **J.L.:** conceptualization (lead), data curation (supporting), formal analysis (equal), writing – original draft (lead).

## Conflicts of Interest

The authors declare no conflicts of interest.

## Data Availability

The group‐level SPMs (related to Figure 2A) and the PEB and BMA files (related to Figure 2B and Figure 3) are available under: https://doi.org/10.5281/zenodo.17953854. These data can be inspected using the SPM package (https://www.fil.ion.ucl.ac.uk/spm), which also includes the open‐source code for the DCM analyses. The single‐participant data cannot be shared because of privacy/ethical restrictions.
